# Individual Differences in Colour Perception: The Role of Low-Saturated and Complementary Colours in Ambiguous Images

**DOI:** 10.1177/20416695211055767

**Published:** 2021-11-26

**Authors:** EunYoung Jeong, In-Ho Jeong

**Affiliations:** School of Chemical Engineering, 92201 Sungkyunkwan University, Gyeonggi-do, Republic of Korea; Department of Neurosurgery, Miraero 21 Medical Center, Gwangju Republic of Korea

**Keywords:** individual differences, colour perception, colour constancy, illumination, complementary colours, low-saturated colour, #thedress

## Abstract

Individual differences in colour perception, as evidenced by the popular debate of “The Dress” picture, have garnered additional interest with the popularisation of additional, similar photographs. We investigated which colorimetric characteristics were responsible for individual differences in colour perception. All objects of the controversial photographs are composed of two representative colours, which are low in saturation and are either complementary to each other or reminiscent of complementary colours. Due to these colorimetric characteristics, we suggest that one of the two complementary pixel clusters should be estimated as the illuminant hue depending on assumed brightness. Thus, people perceive the object's colours as being biased toward complementarily different colour directions and perceive different pixel clusters as chromatic and achromatic. Even though the distance between colours that people perceive differently is small in colour space, people perceive the object's colour as differently categorized colours in these ambiguous photographs, thereby causing debate. We suggest that people perceive the object's colours using different “modes of colour appearance” between surface-colour and self-luminous modes.

## Introduction

When an image of a two-coloured dress was posted online in February 2015, it went viral worldwide because people strongly disagreed about whether “The Dress” was blue and black, or white and gold in colour. A few people perceived both colours as blue and gold, both of which were chromatic. The most striking result of these debates is that individuals can perceive different colours in an identical image (Brainard & Hurlbert, 2015; Conway, 2015; Gegenfurtner et al., 2015; Lafer-Sousa et al., 2015; The dress, n.d.; Witzel et al., 2017a). This topic garnered great social media attention, with many tweets mentioning “The Dress” debate, with hashtags such as #thedress, #dressdebate, and #dressgate.

Many studies have shown that people differ not only in how they name “The Dress” colour but also in the chromaticity to which they match it (Aston & Hurlbert, 2017; Gegenfurtner et al., 2015; Lafer-Sousa et al., 2015; Witzel et al., 2017a). Clearly, there are significant individual differences in the processes of colour perception; many authors emphasized that the colour constancy hypothesis accounts for the individual differences in colour perception (Aston & Hurlbert, 2017; Brainard & Hurlbert, 2015; Conway, 2015; Gegenfurtner et al., 2015; Hugrass et al., 2017; Lafer-Sousa et al., 2015; Lafer-Sousa & Conway, 2017; Toscani et al., 2017; Uchikawa et al., 2017; Wallisch, 2017; Witzel et al., 2017a; Witzel et al., 2017b). The idea is that individual differences in colour perception arise because people make different estimations about the illuminant colours in the scene (Aston & Hurlbert, 2017; Brainard & Hurlbert, 2015). Moreover, many authors have reported that individual differences in colour perception of “The Dress” arose because viewers made different estimations about a given scene's incident illumination qualities as yellow or blue (Aston & Hurlbert, 2017; Brainard & Hurlbert, 2015; Conway, 2015; Hugrass et al., 2017; Lafer-Sousa et al., 2015; Lafer-Sousa & Conway, 2017; Wallisch, 2017; Witzel et al., 2017a; Witzel et al., 2017b). To prove the idea that illumination estimations underlie the individual differences in colour perception of “The Dress”, Lafer-Sousa et al. (2015) cut “The Dress” from the original photograph and embedded it in scenes containing unambiguous cues pointing to either a yellow or blue illumination. As the cues to the illumination are enhanced, most people conformed to a single categorical percept consistent with the illumination cued (Lafer-Sousa et al., 2015; Lafer-Sousa & Conway, 2017). However, there remains the question about what colorimetric characteristics of the picture result in individual differences in the estimation of illumination.

In this study, we showed that individual differences in colour perception are due to the pixel colour characteristics that have not yet been reported. That is, the pixel colours have a low saturation throughout the surface of the object and are divided into two complementary colour clusters: low-saturated yellow and low-saturated blue. In the absence of any information about the illuminant in a scene, a common mechanism for achieving colour constancy is the approximate assumption of the spatial average of the scene reflectance to be the illuminant colour (Brainard, 1998; Foster, 2011; Granzier et al., 2009). However, in the ambiguous photograph of “The Dress”, there is no common colour distribution in the scene, as both colour stripes of “The Dress” complement each other. The pixel colours are separated into two colour categories that can both be estimated as the illuminant in the scene. The general gray world hypothesis is not helpful in estimating the illuminant in this photograph. When at least one pixel cluster of “The Dress” is high in saturation, the variability in colour perception will be minimal even in the absence of any information on the illuminant (Foster, 2011). However, the pixel colours in “The Dress” were constantly low in saturation, and the absolute International Commission on Illumination (CIE) a* and b* values of the object's pixels were under 35 when we checked. Pixels with low saturation values tend to contain more complicated information related to the illuminant than the highly saturated colours (Ahn et al., 2013; Morimoto et al., 2016).

Given such difficulties in estimating the illuminant in the scene, we propose that viewers are forced to estimate one of two complementary colours as an illuminant hue. Considering that the visual system maintains colour constancy by setting the estimated illuminant as the neutral point (Brainard, 1998; Brown & MacLeod, 1997; Conway, 2009; Delahunt & Brainard, 2004; Foster, 2011), individual differences in achromatic colour (white or black) perception mean that viewers might interpret different chromatic distributions in “The Dress” photograph as evidence of the illuminant (Brainard & Hurlbert, 2015; Lafer-Sousa et al., 2015; Witzel et al., 2017b). Moreover, it has been reported that the chromaticity signals of an estimated illuminant result in a complementary shift in the perceived colour of the object (Brown & MacLeod, 1997; Conway, 2009; Foster, 2011; Judd, 1940). Thus, depending on which of the two complementary colours is estimated to be the illuminant, people necessarily perceive “The Dress” colours as being biased toward complementarily different colour directions. For those who estimated the low-saturated yellow pixel cluster as a reference chromaticity for illuminant in the scene, they perceived “The Dress” colours as being biased toward the blue direction, which is the complementary colour to the estimated illuminant. The low-saturated yellow lost saturation and was perceived as an achromatic colour, black. Furthermore, the low-saturated blue got saturated and was perceived as a more saturated blue. In contrast, those who estimated the illuminant as the low-saturated blue, perceived “The Dress” colours as being biased toward the yellow direction. The low-saturated yellow was perceived as a more saturated chromatic colour, gold, and the low-saturated blue was perceived as an achromatic colour, white. Thus, the stripe of “The Dress” that some people perceived as chromatic was perceived as achromatic by other people and vice versa. In addition, the differently perceived chromatic colours were complementary to each other, and had little in common. In general, individual differences in colour perception according to the estimation of the illuminant are reflected in changes of saturation in the same colour hue (Judd, 1940). However, in “The Dress”, the differences are shown not only as changes in saturation but also as changes in colour category between chromatic and achromatic categories. Therefore, the individual difference in colour naming was prominent as people perceived the object's colours as differently categorized hues, even though the distance between differently perceived colours among people was minimal in colour space.

Furthermore, it has been reported that the subjective assumption of the brightness in the scene can lead to individual differences in the choice of illuminant colours, and eventually to individual differences in the perceived colour of “The Dress” (Aston & Hurlbert, 2017; Gegenfurtner et al., 2015; Hugrass et al., 2017; Morimoto et al., 2021; Toscani et al., 2017; Uchikawa et al., 2017; Witzel et al., 2017a). Brightness in the same scene can be assumed differently, as there are large and reliable individual differences in subjective luminance sensitivity (Brainard & Hurlbert, 2015; Mollon et al., 2017), and a surprisingly large range of chromaticity can be perceived as white by different observers and even the same observer on different occasions (Bosten et al., 2015; Judd, 1940; Mollon et al.*,* 2017). Different assumptions about the brightness in the same scene could explain the distinct difference in lightness perception of “The Dress” among people. When the perceived lightness was high, people tended to perceive “The Dress” colours as white and yellow; the lightness was low in the blue and black group (Gegenfurtner et al., 2015). Even changing the apparent brightness could alter the chromatic perception of “The Dress” (Hugrass et al., 2017). Perceptual dimming shifted the colour categorization toward blue and black, whereas perceptual brightening shifted the colour naming toward white and gold (Hugrass et al., 2017). To explain how the subjective assumptions of brightness showed an opposite trend to the illumination matches in this ambiguous photograph, Uchikawa's group (Morimoto et al., 2021; Uchikawa et al., 2017) showed that the optimal colour hypothesis predicts a discrepancy in the estimated illuminant colour dependent on the assumed brightness. When the brightness was assumed to be low, a high colour temperature was estimated as illuminant, assuming a high brightness led to the estimation of a low colour temperature. In addition, Witzel et al. (2017a) explained that people could estimate the illuminants in the scene of “The Dress” photograph by considering the natural daylight locus. Generally, the illumination colour is optimized to a cold colour when there is an object in the shadow or indirect lighting. In contrast, a warm colour is optimized as the illuminant colour when the object is exposed to direct light under the sun. Thus, those who assume the brightness in the scene is low might consider “The Dress” as being in the shadow or indirect lighting, which is linked to the estimation of darker and colder illumination, and they perceive its colours to be white and yellow. On the contrary, those who assume high brightness in the scene, might interpret “The Dress” as being under the direct light of the sun, which is linked to the estimation of a brighter and warmer illuminant, and they perceive its colour as blue and black (Aston & Hurlbert, 2017; Gegenfurtner et al., 2015; Hugrass et al., 2017; Toscani et al., 2017; Uchikawa et al., 2017; Witzel et al., 2017a). However, it should be assessed whether this explanation is applicable to other photographs that are also characterized by personal differences in colour perception.

A few people perceived the colour of “The Dress” as gold and blue, which are both chromatic. We think that they relied on information about chromaticity, rather than brightness, to perceive colours in this ambiguous photograph. In general, when two colours are complementary to each other, people usually perceive the colours using colour contrast. In addition, colour contrast is expressed maximally when the brightness contrast between two colours is small (Brown & MacLeod, 1997). Therefore, if these people perceive the object's colours through colour contrast, the chromaticity distance between two perceived colours in this group is expected to be larger than that of the others, and the lightness distance between two perceived colours is expected to be smaller than the others.

Recently, photographs similar to “The Dress” have appeared in popular literature, thus once again bringing this issue of individual differences in colour perception to the forefront of public discussion. Using the International Commission on Illumination (CIE) (L*, a*, b*) (CIELAB) colour space, we will check whether the pixel clusters of each subject are low saturated and complementary to each other, as well as whether there is a consistent characteristic in the way individual differences occur in colour perception.

## Method

### Survey Object Selection

We conducted online searches for “what colour is it”, “white and gold”, “blue and black”, “#thedress”, “dress gate”, and “dress debate”. Using a Google Chrome browser, we tried to find photographs containing images of two-coloured objects, where perceptual differences were common to both colours. If differences in perception occurred in only one colour, the photograph was excluded. We identified five photographs that met the above criteria (Alisha, 2017; Arthur, 2017; Mariam1a, 2016; Rafaella, 2017; The dress, n.d.).

### Colorimetric characteristic assessment

The International Commission on Illumination (CIE) (L*, a*, b*) (CIELAB) colour space, defined in 1976, was used for all analyses. This colour space is known to closely mimic human colour perception (CIELAB colour space, n.d.). To obtain CIELAB values for both pixel clusters of each object, we sampled five different points in each colour area of the object, and the mean values were recorded. We checked whether both pixel clusters in any object were weakly saturated. Additionally, we tested whether the two pixel clusters in any given object were related in a complementary manner by illustrating the mean CIE a* and b* values of the two pixel clusters along the CIE (a* and b*) planes. If the two pixel clusters were complementary to each other, pairs might face each other toward the center of the graphs (Pridmore, 2009; Pridmore, 2011; Westland et al., 2007).

### Preparation of Colour Palettes for Survey

From the measured mean CIELAB values of the pixel colours, new colour values were obtained by adding and subtracting 7, 14, and 21 in each of the L*, a*, and b* values, and 343 new colour values were obtained for each pixel cluster. Colour palettes with 343 different variants for each pixel cluster were prepared. In the process of preparing colour palettes, if L* values were over 100, 100 was used because 100 is the upper limit of the L* values.

### Survey Progress

Between 20 February to 20 March 2020, a survey was conducted for 90 people comprising 61 women and 29 men. The average participant age was 29.76 (13–51) years. Participants were Korean in origin, and surveys were conducted in the Korean language. All participants had normal visual acuity and no colour vision deficiencies.

The stimuli were presented on a light emitting diode (LED) monitor (Samsung S19E450, 19 in., 1280 × 1024 pixels; Samsung, Suzhou, China). The monitor was controlled using a 32-bit Windows machine, equipped with a graphic card (Intel HD Graphics 4400; Santa Clara, CA) and a refresh rate of 59 Hz. Instructions were presented in the colour of the monitor white point (CIExyY; 0.3145, 0.3247, 81 cd/m^2^). The photographs were presented in the center of the white screen. The image sizes were 20–21° × 14–21° visual angle (Case 1, 20° × 14°; Case 2, 21° × 14°; Case 3, 20° × 21°; Case 4; 20° × 15°; and Case 5, 20° × 16°). Participants sat 60 cm from the center of the computer screen with their head stabilised by a head rest. After viewing each prepared photograph for more than 5 s, we asked respondents to name the colours of the object in each photograph.

After the colour-naming task, we showed participants a new screen in which one pixel cluster of the original object was marked with an arrow, and the prepared colour palettes were displayed on the same screen. Each matching sample in the palettes was the same size on all trials (1.91° visual angle). We asked respondents to choose the most similar colour in the prepared colour palettes to the perceived colour. If there were no similar colours in the prepared colour palettes, we asked them to find matched colours using the Adobe Colour Picker tool. When the perceived colour changed during the experiment, we asked them to conduct the experiment again starting from the beginning, and we selected the results for the last colour perception. In the same way, colour matching was performed in the other pixel cluster. The experiments in each case took about 7 min. All colour matches were converted to CIELAB values for analysis.

### Classification of the Respondents According to Colour Perception

Most participants perceived two colours of each object as a pair of achromatic and chromatic colours. They were classified into groups 1 and 2 according to the perceived lightness of the achromatic colour. Those who perceived one pixel cluster as bright achromatic, such as white, light gray, or silver, and the other pixel cluster as chromatic were classified as group 1. Those who perceived one pixel cluster as dark achromatic, such as gray or black, and the other cluster as chromatic were classified as group 2. Those who perceived both pixel clusters as chromatic were classified as group 3.

### Assessing the Perceived Colour Difference among Groups

To check the distribution of perceived chromaticity, we plotted the colour matches of all respondents on the a* and b* graphs, after which we plotted the average colour matches perceived by each group. Through this, we could visually show that there was a difference in colour perception among people. In addition, we checked which of the two complementary colours was perceived as achromatic and the direction toward which the colours were perceived as being biased.

To check whether there is a difference in the perceived lightness of the object among people, we expressed the L* value perceived in each group as a 95% confidence interval on the L* graphs. Through this, we were able to demonstrate that there was a difference in the lightness perceived in groups 1 and 2.

### Statistical Exploration of the Perception Difference Between Groups 1 and 2

To demonstrate that there is a difference in perceived lightness and chromaticity between groups 1 and 2, we used the Wilcoxon signed-rank test. We compared each of the CIE L*, a*, and b* values between two groups. When differences in the same value or values are commonly observed in both pixel clusters, we judged that there was a difference in colour perception between two groups, in the colour parameter corresponding to that value or those values.

In addition, we checked the pairwise Pearson's correlation coefficient between the perceived lightness (L* values) and the perceived chromaticity (a* and b* values) to check whether the perceived chromaticity might depend on the assumed brightness.

### Characteristics of Colour Perception in Group 3

Assuming that group 3 perceived the object's colours by colour contrast, we expected that there would be a larger chromaticity distance (ΔC*) between two perceived colours than that of the other groups, and a smaller lightness distance (ΔL*) between two perceived colours. To confirm this, we performed a Kruskal–Wallis analysis on Δ*C* * and ΔL* among the three groups, and a Bonferroni verification was performed as a post-hoc test.

The formulas were as follows:

$$\begin{array}{rl} & \Delta C*=\sqrt{{\left(a1-a2\right)}^{2}+{\left(b1-b2\right)}^{2}}\\ & \Delta L*=\;L1\;-\;L2 \end{array}$$

a1, b1, and L1 are the CIELAB values of the brighter pixel cluster of each object, and a2, b2, and L2 are the values of the darker pixel cluster.

All analyses of this study were verified at a significance level of *p* < .05. The software Statistical Package for the Social Sciences for Windows, Version 24.0 (SPSS/WIN 24.0) was used for statistical processing. This study was carried out in accordance with the Declaration of Helsinki, and the protocol was approved by the Ethics Committee of Sungkyunkwan University.

## Results

### Case 1

**Figure 1.**
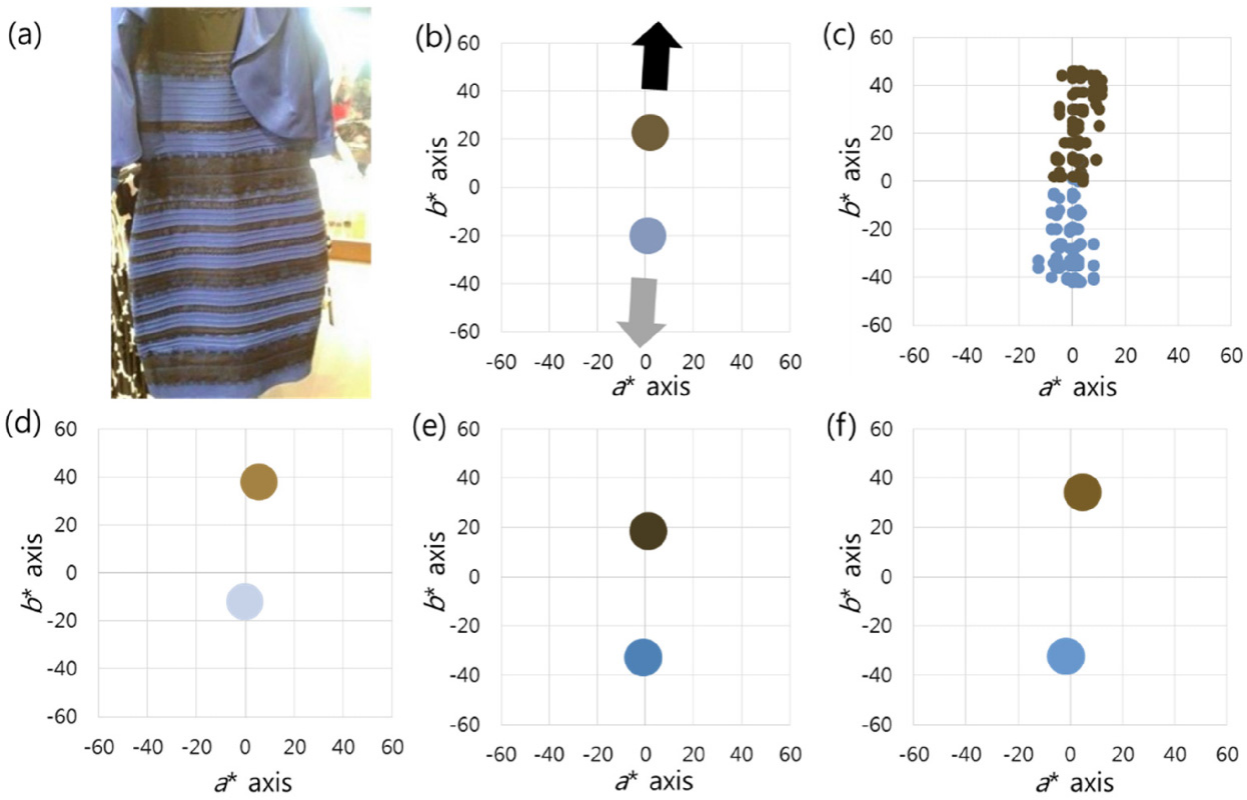
(a) This is the first photograph that elicited public attention to individual differences in colour perception. (b) Both pixel clusters are low in saturation, and they are complementary to each other. The low-saturated blue is brighter than the low-saturated yellow. The black arrow indicates the darker cluster direction, and the light gray arrow indicates the brighter cluster direction. (c) When the colour matches of all respondents are displayed, there is a perception difference along the b* axis. (d) In group 1, both pixel clusters are perceived as being biased in the (+) direction of the b* axis, the darker pixel direction. (e) In group 2, both pixel clusters are perceived as being biased in the (−) direction of the b* axis, the brighter pixel direction. (f) In group 3, the two pixel clusters are biased in opposite directions of the b* axis. The average values are drawn in batches on the graphs. *Photograph of “The Dress’ is used with kind permission. Copyright Cecilia Bleasdale.

Case 1 is the starting point of this topic, the picture of “The Dress” (The dress, n.d.). The object consisted of two pixel clusters, low-saturated blue and low-saturated yellow (Figure 1a). The former was brighter than the latter, and the mean CIELAB values of each colour stripe of “The Dress” were (63, 1, −20) and (41, 2, 23), respectively. They were complementary to each other by facing each other toward the center of the CIE a* and b* planes (Figure 1b). When the colour matches of all respondents were displayed on the a* and b* planes, there was a continuous distribution of colour percepts along the b* axis (Figure 1c). The mean values were (59.58, −0.94, −28.63) and (33.3, 2.39, 23.86). A total of 18 (20%) people perceived the colours of “The Dress” as white and gold and were classified as group 1. The perceived achromatic colour was subdivided into light gray, silver, and white. The perceived chromatic colour was subdivided into yellow and gold. Group 1 perceived the object's colours as being skewed from the original pixels in the (+) direction of b* axis, the darker pixel (low-saturated yellow) direction (Figure 1d). The brighter cluster (low-saturated blue) was perceived as achromatic (white), and the darker cluster (low-saturated yellow) was perceived as chromatic (gold). There were 64 (71.11%) people who perceived the colour of “The Dress” as blue and black, and they were classified as group 2. Group 2 perceived both pixel clusters as being biased in the (−) direction of the b* axis, the brighter pixel (low-saturated blue) direction (Figure 1e). The brighter cluster (low-saturated blue) was perceived as chromatic (blue), and the darker cluster (low-saturated yellow) was perceived as achromatic (black). A total of eight (8.9%) people perceived both pixel clusters as chromatic, such as blue and gold, or blue and purple and were classified as group 3. The average CIELAB values were (61.25, −1.63, −32.25) and (41, 4.63, 34.38), respectively. The two pixel clusters were perceived as being biased in opposite directions of the b* axis (Figure 1f).

**Figure 2.**
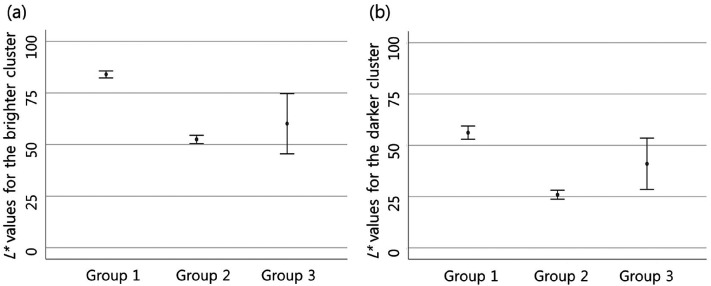
In Case 1, there is a difference in the object's lightness perceived between groups 1 and 2. Group 1 perceived the object's lightness as higher than group 2 in both pixel clusters (in both clusters, *p *< .001). The perceived lightness for the brighter pixel cluster (a) and for the darker pixel cluster (b) is expressed by calculating a 95% confidence interval. In group 3 there is a wider 95% confidence interval as a small number of participants were classified as this group.

There was also a difference in the perceived lightness of the object among groups. The difference was significant between groups 1 and 2. Group 1 perceived the object's lightness as higher than group 2, and it was statistically significant in both pixel clusters (Figure 2, Table 1).

**Table 1.** The Statistical Results of the Perception Differences Between groups 1 and 2.

		Brighter Pixel Cluster			Darker Pixel Cluster		
		L* value	a* value	b* value	L* value	a* value	b* value
Case 1	Group 1	84 ± 3.4	−0.2 ± 4.3	−11.8 ± 10.8	56.2 ± 3.6	5.5 ± 3.6	37.8 ± 7.2
	Group 2	52.5 ± 8.1	−1.1 ± 3.9	−32.9 ± 7.2	25.9 ± 8.7	1.2 ± 3.6	18.6 ± 14.5
	*p* value	<.001	.475	<.001	<.001	<.001	<.001
Case 2	Group 1	91.7 ± 4.5	−3.4 ± 6	−4.9 ± 8.7	61.6 ± 6.6	3.4 ± 3.9	28.7 ± 5.8
	Group 2	70.1 ± 8.7	−6.1 ± 4.2	−31.2 ± 6.3	46.6 ± 11.9	0 ± 3.8	6.1 ± 10.9
	*p* value	<.001	.008	<.001	<.001	.003	<.001
Case 3	Group 1	98.1 ± 3.2	−8.6 ± 8.5	−3.2 ± 6	83.4 ± 3.8	11.9 ± 3.6	−0.2 ± 5.5
	Group 2	91.2 ± 7.1	−30.1 ± 7.8	−4.3 ± 5.5	67.9 ± 9.5	−2.3 ± 4.2	−1 ± 4
	*p* value	<.001	<.001	.530	<.001	<.001	.421
Case 4	Group 1	98.3 ± 4	−5.1 ± 8.1	−8 ± 7.9	64.2 ± 5.9	19.4 ± 6.5	1.5 ± 5.5
	Group 2	91.7 ± 6.9	−26.5 ± 7.1	−9.9 ± 5.7	58.2 ± 11.4	0.8 ± 4.4	1.7 ± 4.3
	*p* value	<.001	<.001	.284	.026	<.001	.840
Case 5	Group 1	80.3 ± 11.9	−0.2 ± 2.9	5.7 ± 4.7	52.3 ± 9.4	−13.9 ± 5.7	−10.4 ± 5.8
	Group 2	51.5 ± 12.1	6.5 ± 5.1	22 ± 6.6	25.5 ± 6.4	−6 ± 6.1	−1 ± 7.1
	*p* value	<.001	<.001	<.001	<.001	<.001	<.001

In all cases, there is a difference in the perceived lightness of the object between groups 1 and 2, in both pixel clusters. Also, there is a difference in the object's chromaticity perceived between the two groups. In Cases 1 and 2, there is a difference primarily in the b* values. In Case 2, the difference is also found in a* values. In Cases 3 and 4, the difference is found in the a* values. In Case 5, differences in both a* and b* values are found.

### Case 2

**Figure 3.**
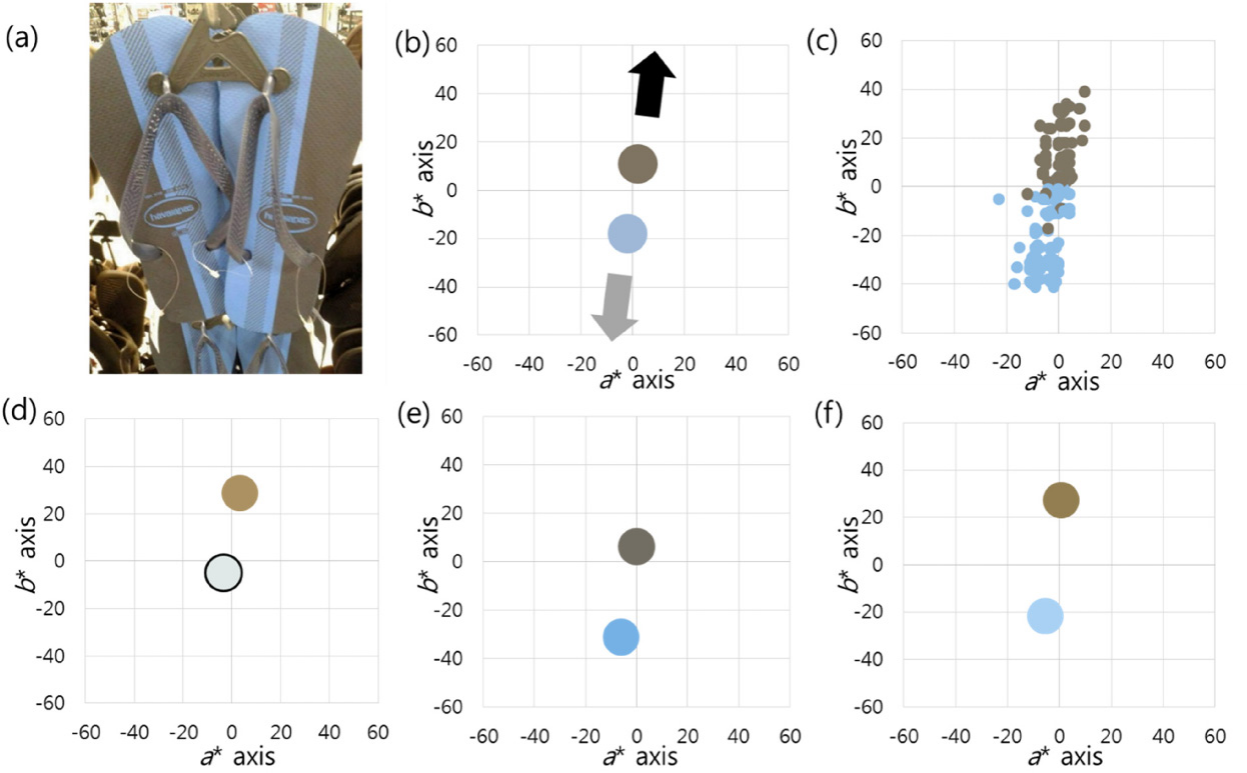
(a) This “flip-flops’ photograph causes a difference in colour perception among people. (b) Both pixel clusters are low saturated and complementary to each other. The low-saturated blue is brighter than the low-saturated yellow. The black arrow indicates the darker cluster direction, and the light gray arrow indicates the brighter cluster direction. (c) There is a continuous distribution of colour percepts along the b* axis when the colour matches of all respondents are displayed. (d) In group 1, both pixel clusters are perceived as being biased in the (+) direction of the b* axis, the darker pixel direction. (e) In group 2, both pixel clusters are perceived as being biased in the (−) direction of the b* axis, the brighter pixel direction. (f) In group 3, the two pixel clusters are biased in opposite directions of the b* axis. The average values are drawn in batches on the graphs.

Case 2 corresponds to a picture of a pair of flip-flops that consist of two pixel clusters, low-saturated blue and low-saturated yellow (Arthur, 2017; Figure 3a). The mean CIELAB results of the two pixel clusters were (74, −2, −18) and (49, 2, 11), respectively. The low-saturated blue was brighter than the low-saturated yellow. They were complementary to each other (Figure 3b). When the colour matches in all respondents were displayed, there was a continuous distribution of colour percepts along the b* axis (Figure 3c). The mean values of each perceived colour were (74.75, −5.58, −26.17) and (49.54, 0.6, 11.31). In group 1, there were 15 (16.67%) people who perceived the colours of the object as white and yellow. The perceived achromatic colour, white, was subdivided into light gray, silver, and white. They perceived both pixel clusters as being biased in the (+) direction of b* axis, the darker pixel (low-saturated yellow) direction (Figure 3d). The brighter cluster (low-saturated blue) was perceived as achromatic (white), and the darker cluster (low-saturated yellow) as chromatic (yellow). In group 2, 69 (76.67%) people perceived the object's colours as blue and black. The perceived achromatic colour, black, was subdivided into dark gray and black. People perceived both pixel clusters as being biased in the (−) direction of the b* axis, the brighter pixel (low-saturated blue) direction (Figure 3e). The brighter cluster (low-saturated blue) was perceived as chromatic (blue), and the darker cluster (low-saturated yellow) as achromatic (black). In group 3, six (6.67%) people perceived the colours as blue and gold. The two pixel clusters were perceived as being biased in opposite directions of the b* axis (Figure 3f).

**Figure 4.**
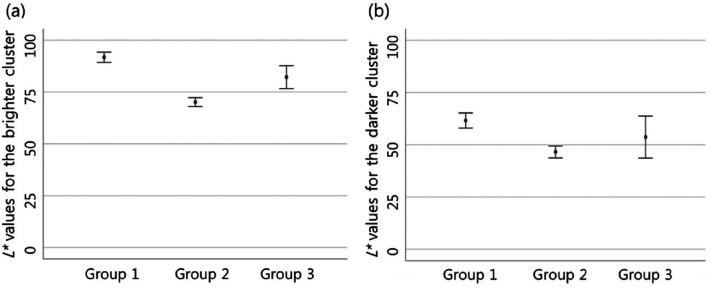
In Case 2, group 1 perceived the object's lightness as higher than group 2 in both pixel clusters (in both clusters, *p* < .001). The lightness perceived for the brighter pixel cluster (a) and for the darker pixel cluster (b) are expressed by calculating a 95% confidence interval. In group 3, there is a wider 95% confidence interval as a small number of participants were classified as group 3.

There was also a difference in the perceived lightness of the object among groups. The difference was significant between groups 1 and 2. Group 1 perceived the object's lightness as higher than group 2, and it was statistically significant in both pixel clusters (Figure 4, Table 1).

### Case 3

**Figure 5.**
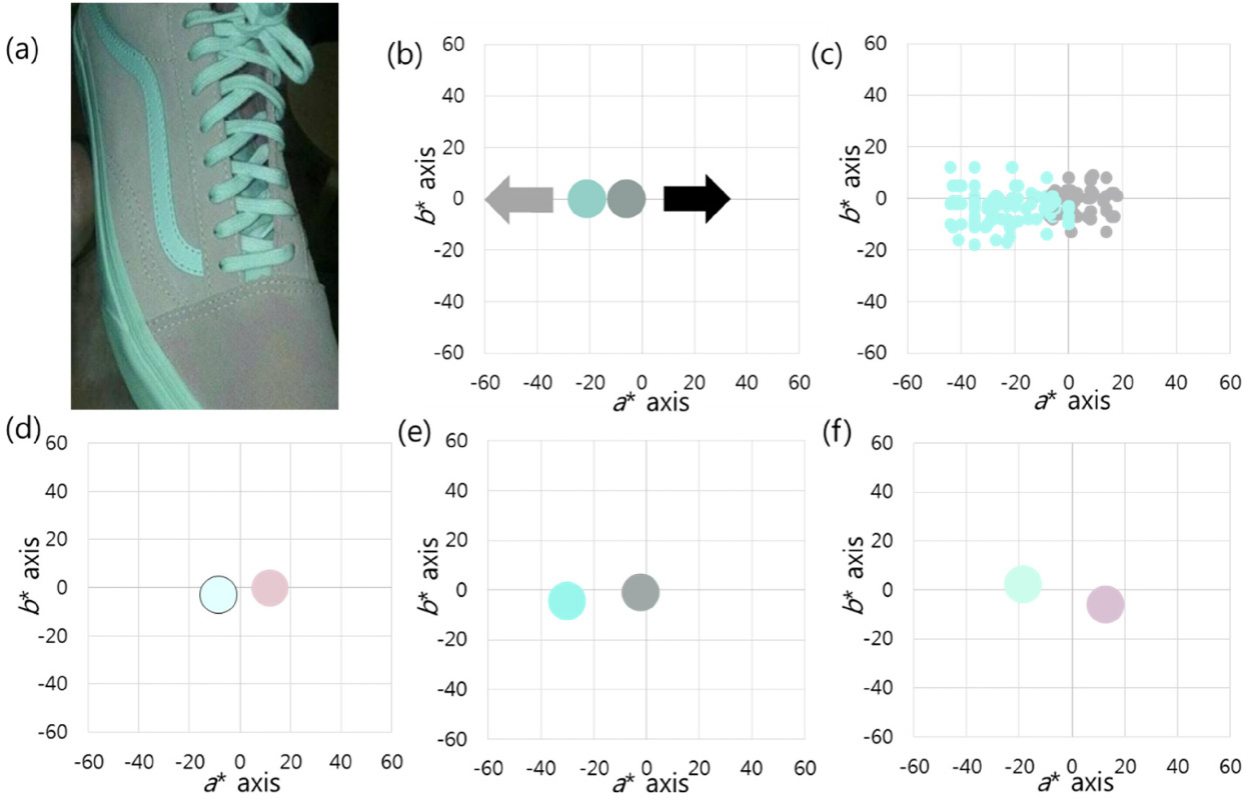
(a) There is a perception difference among people between teal and pink in this shoe photograph. (b) The object consists of two pixel clusters, low-saturated green and more desaturated green. The former is brighter than the latter. The black arrow indicates the darker cluster direction, and the light gray, the brighter cluster direction. They are not complementary, but the line connecting the two pixel clusters passes through the center of the graphs. (c) When the colour matches of all respondents are displayed, there is a continuous distribution of colour percepts near the a* axis. (d) In group 1, both pixel clusters are perceived as being biased in the (+) direction of the a* axis. (e) In group 2, the low-saturated green is biased in the (−) direction of the a* axis, and the more desaturated green is biased to the center of the graphs. (f) In group 3, the low-saturated green is located in the (−) area of the a* axis, but the more desaturated green is located in the (+) area of the a* axis. The average values are drawn in batches on the graphs.

In Case 3, a shoe and a part of the hand were seen with a dark background (Alisha, 2017; Figure 5a). During the survey, seven of the participants switched their perception of the image colours spontaneously. We chose the final colour perception for this study. The object consisted of two pixel clusters, low-saturated green and more desaturated green, which could be considered almost as gray. The former was brighter than the latter. We checked the CIELAB values only on the side of the shoe; the mean values were (79, −21, −2) and (64, −6, 0), respectively. The two pixel clusters were not complementary to each other, as they did not face the center of the graphs, but the line connecting the two colours passed through the center (Figure 5b). When the colour matches of all respondents were displayed, there was a continuous distribution of colour percepts along the a* axis (Figure 5c), and the mean values were (92.68, −25.42, −3.83) and (71.43, 1.07, −0.98), respectively. In group 1, 18 (20%) people perceived the colours of the shoe as white and pink. They perceived both pixel clusters as being biased in the (+) direction of the a* axis, the darker pixel (more desaturated green) direction (Figure 5d). The brighter cluster (low-saturated green) was perceived as achromatic (white), and the darker cluster (more desaturated green) was perceived as chromatic (pink). In group 2, 69 (76.67%) people perceived the colours as teal and gray (Figure 5e). The brighter cluster (low-saturated green) was perceived as a more saturated colour, teal, by being biased toward the (−) direction of the a* axis. The darker cluster (more desaturated green) was perceived as achromatic gray by being biased toward the (+) direction of the a* axis and was located near the center of the graphs (Figure 5e). In group 3, three (3.33%) people perceived both pixel clusters as chromatic, teal, and pink. The brighter cluster was perceived to be similar to the pixel colours by a slight deflection in the (+) direction along the a* axis. The darker cluster was more biased along the a* axis and was located in the (+) area of the a* axis (Figure 5f).

**Figure 6.**
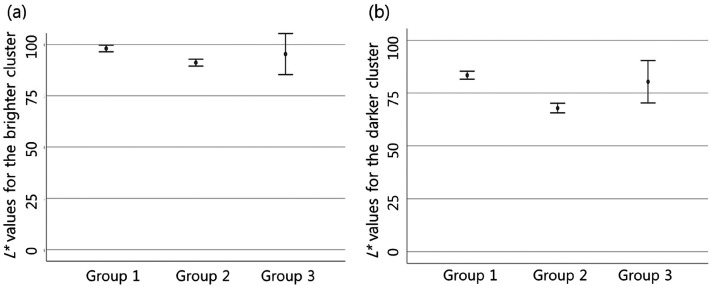
In Case 3, group 1 people perceived the object's lightness as higher than group 2 in both pixel clusters (in both clusters, *p* < .001). The perceived lightness for the brighter pixel cluster (a) and for the darker pixel cluster (b) is expressed by calculating a 95% confidence interval. In group 3, there is a wider 95% confidence interval as a small number of participants were classified as this group.

In addition, there was a difference in the perceived lightness of the object among groups. The difference was significant between groups 1 and 2. Group 1 perceived the object's lightness as higher than group 2, and it was statistically significant in both pixel clusters (Figure 6, Table 1).

### Case 4

**Figure 7.**
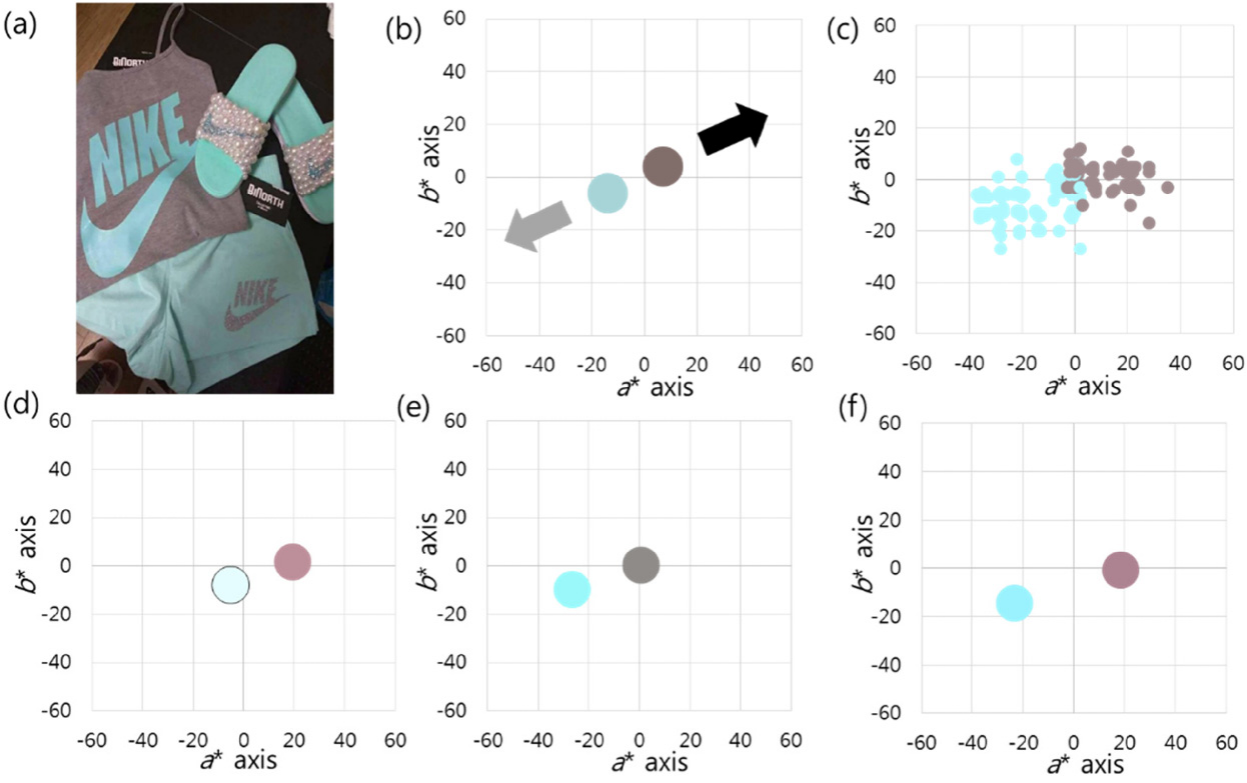
(a) A picture of a tank top, pants, and slippers; in this study, the tank top is the target object. (b) Both pixel clusters are low saturated and complementary to each other. The low-saturated blue–green is brighter than the low-saturated yellow–red. The black arrow indicates the darker cluster direction, and the light gray arrow indicates the brighter cluster direction. (c) There is a continuous distribution of colour percepts near the a* axis, when the colour matches of all respondents are displayed. (d) In group 1, both pixel clusters are perceived as being biased in the (+) direction of the a* axis, which is almost the darker pixel direction. (e) In group 2, both pixel clusters are perceived as being biased in the (−) direction of the a* axis, almost the brighter pixel direction. (f) In group 3, the two pixel clusters are biased in the opposite directions of the a* axis. The average values are drawn in batches on the graphs.

Case 4 corresponds to a picture of a tank top, pants, and slippers (Rafaella, 2017; Figure 7a). We targeted only the tank top among the subjects in the picture. The tank top consisted of two pixel clusters, low-saturated blue–green and low-saturated yellow–red. The former was brighter than the latter. The mean CIELAB values were (82, −14, −6) and (48, 7, 4), respectively. Both pixel clusters faced each other through the center of the graphs (Figure 7b). The colour matches of all participants were continuous near the a* axis (Figure 7c). The mean values were (93.13, −20.77, −10.13) and (59.82, 7.86, 1.28), respectively. In group 1, 22 (24.44%) people perceived the colours as white and pink. They perceived both pixel clusters as being biased to the (+) direction of a* axis, which is almost the darker pixel direction (Figure 7d). The brighter cluster (low-saturated blue–green) was perceived as achromatic (white), and the darker cluster (low-saturated yellow–red) was perceived as chromatic (pink). In group 2, 55 (61.11%) people perceived the colours as turquoise and gray. They perceived both pixel clusters as being biased to the (−) direction of the a* axis, almost the brighter pixel direction (Figure 7e). The brighter cluster (low-saturated blue–green) was perceived as chromatic (turquoise), and the darker cluster (low-saturated yellow–red) was perceived as achromatic (gray). In group 3, 13 (14.44%) people perceived the colours as turquoise and pink. Two low-saturated pixel clusters were perceived as being biased in opposite directions of the a* axis (Figure 7f).

**Figure 8.**
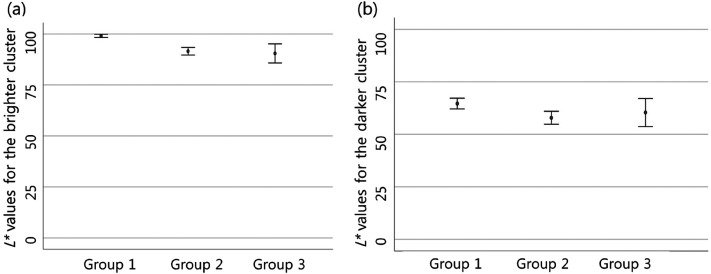
In Case 4, group 1 perceived the object's lightness as higher than group 2 in both pixel clusters (in brighter cluster, *p* < .001; in darker cluster, *p* = .026). The lightness perceived for the brighter pixel cluster (a) and for the darker pixel cluster (b) is expressed by calculating a 95% confidence interval. In group 3, there is a wider 95% confidence interval as a small number of participants were classified into this group.

There was also a difference in the perceived lightness of the object among groups. The difference was significant between groups 1 and 2. Group 1 perceived the object's lightness as higher than group 2, and it was statistically significant in both pixel clusters (Figure 8, Table 1).

### Case 5

**Figure 9.**
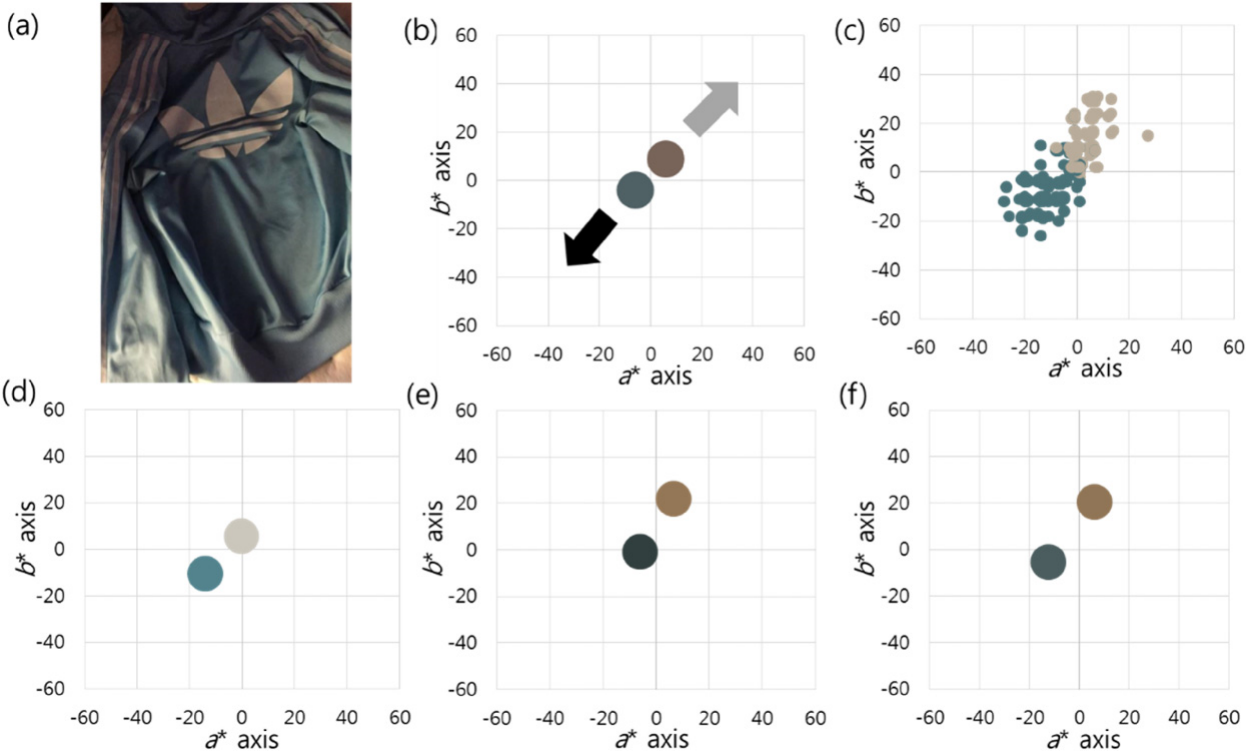
(a) A picture of the shirt regarding which there is a colour perception difference among people. (b) The shirt consists of two pixel clusters, low-saturated yellow–red and low-saturated blue–green. The former is brighter than the latter. They are complementary to each other. The black arrow indicates the darker cluster direction, and the light gray arrow indicates the brighter cluster direction. (c) When the colour matches of all respondents are displayed, there is a continuous distribution of colour percepts diagonally against the a* and b* axes. (d) In group 1, the two pixel clusters are perceived as being biased in the (−) direction of both a* and b* axes, the darker pixel direction. (e) In group 2, the two pixel clusters are perceived as being biased in the (+) direction of both a* and b* axes, the brighter pixel direction. (f) In group 3, the two pixel clusters are biased in opposite directions of both a* and b* axes. The average values are drawn in batches on the graphs.

**Figure 10.**
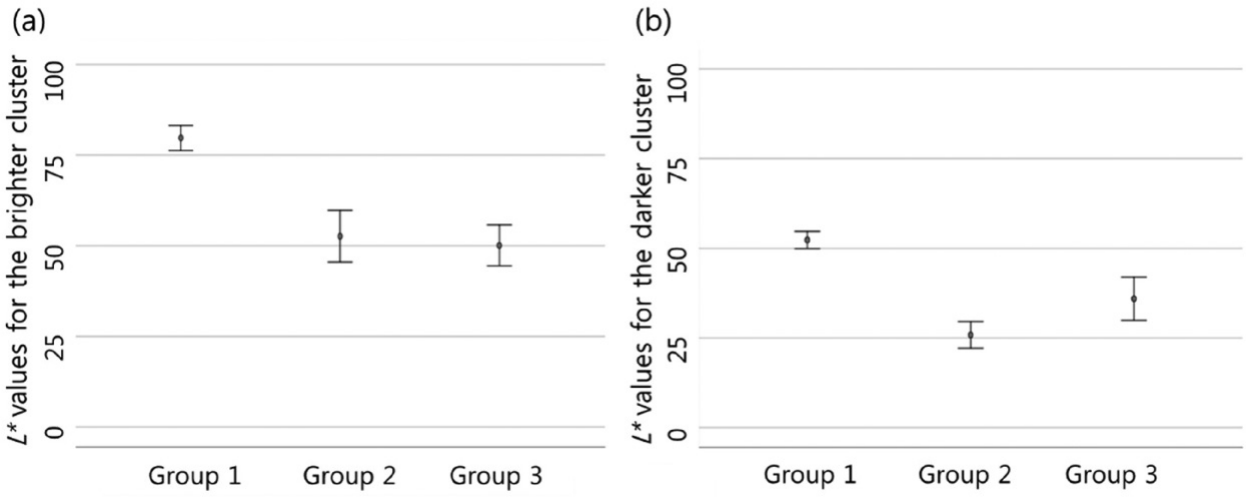
In Case 5, group 1 perceived the object's lightness as higher than group 2 in both pixel clusters (in both cluster, *p* < .001). The lightness perceived for the brighter pixel cluster (a) and for the darker pixel cluster (b) is expressed by calculating a 95% confidence interval.

Case 5 corresponds to the image of a shirt which consists of two pixel clusters, low-saturated yellow–red and low-saturated blue–green (Mariam1a, 2016; Figure 9a). The former was brighter than the latter. The mean CIELAB results were (44, 6, 9) and (40, −6,−4), respectively. They were complementary to each other and faced towards the center of the graphs (Figure 9b). The colour matches of all participants were continuous diagonally against the a* and b* axes (Figure 9c). In group 1, 62 (68.89%) people perceived the colours as white and blue. The perceived achromatic colour, white, was subdivided into light gray, silver, and white. The perceived chromatic colour, blue, was subdivided into light blue, blue, and turquoise. Group 1 perceived both pixel clusters as being biased from the original pixels in the (−) direction of both a* and b* axes, the darker pixel (low-saturated blue–green) direction (Figure 9d). The brighter cluster (low-saturated yellow–red) was perceived as achromatic (white), and the darker cluster (low-saturated blue–green) was perceived as chromatic (blue). In group 2, 14 (15.56%) people perceived the colours as yellow and black. The perceived chromatic colour, yellow, was subdivided into dark yellow, gold, and reddish brown. They perceived both pixel clusters as being biased in the (+) direction of both a* and b* axes, the brighter pixel (low-saturated yellow–red) direction (Figure 9e). The brighter cluster (low-saturated yellow–red) was perceived as chromatic (yellow), and the darker cluster (low-saturated blue–green) was perceived as achromatic (black). In group 3, 14 (15.56%) people perceived the colours as brown and green or gold and green. The two pixel clusters were perceived as being biased in opposite directions of both a* and b* axes, and both pixel clusters were perceived as more saturated chromatic colours (Figure 9f). There was also a difference in the perceived lightness of the object among groups. The difference was significant between groups 1 and 2. Group 1 perceived the object's lightness as higher than group 2, in both pixel clusters (Figure 10, Table 1).

### Perception Differences Between Groups 1 and 2

People in group 1 perceived the object's lightness as higher than those of group 2 in both pixel clusters. In addition, the differences in perceived lightness were statistically significant in all cases (Table 1, Figures 2, 4, 6, 8, and 10). Moreover, groups 1 and 2 perceived the object's colours as being biased from the pixel colours to complementarily different directions. In group 1, the two pixel clusters were perceived as being biased to the darker pixel direction between the two pixel clusters. The brighter cluster was perceived as achromatic and the darker cluster as chromatic. However, in group 2, the two pixel clusters were perceived as being biased toward the brighter pixel direction except in Case 3. In addition, the brighter cluster was perceived as chromatic, and the darker cluster as achromatic. These differences in perceived chromaticity between groups 1 and 2 were statistically significant in all cases. In Cases 1 and 2, the difference in chromaticity perceived between the two groups was statistically significant, mainly along the b* values, yellow–blue distribution. In Case 2, the difference was also found in a* values. In Cases 3 and 4, there was a statistically significant difference in colour perception along the a* values, red–green distribution. In Case 5, in both a* and b* values, it was statistically significant (Table 1).

Furthermore, the correlations between lightness and chromaticity perceived were found in all cases. In Cases 1 and 2, there were positive relationships found between L* and b* values, which were statistically significant in both pixel clusters. In Cases 3 and 4, there were positive relationships revealed between L* and a* values. However, in both cases, it was statistically significant only in one pixel cluster. In Case 5, there were negative relationships between L* and a* values, and between L* and b* values, which were statistically significant in both pixel clusters (Table 2).[Table table2-20416695211055767]

**Table 2. table2-20416695211055767:** Pairwise Pearson's Correlation Between Lightness (L* value) and Chromaticity (a* and b* values) as Perceived in Each Case.

		Brighter Pixel Cluster	Darker Pixel Cluster
		L* value	L* value
Case 1	a* value	0.07	0.42**
	b* value	0.57**	0.55**
Case 2	a* value	0.01	0.27**
	b* value	0.70**	0.24*
Case 3	a* value	0.16	0.57**
	b* value	0.18	0.02
Case 4	a* value	0.41**	0.13
	b* value	0.11	0.18
Case 5	a* value	−0.58**	−0.33*
	b* value	−0.56**	−0.40**

In cases 1 and 2, L* and b* are positively correlated, and the correlation is statistically significant in both pixel clusters. In cases 3 and 4, L* and a* are positively correlated, but the correlation is statistically significant only in one pixel cluster. In Case 5, both L* and a*, and L* and b* are negatively correlated, and the correlation is statistically significant in both pixel clusters. Symbols above the table report significance of Pearson's correlation analysis across observers comparing accuracy to chance level, ***p < .001, **p < .01, *p < 0.05.

### Characteristics of Colour Perception in Group 3

When comparing the results of group 3 with that of groups 1 and 2, ΔL* in group 3 was decreased in cases 1, 4, and 5, but it was statistically significant only in Case 5. ΔC* in group 3 was increased in all cases, but it was statistically significant in Cases 1, 2, and 4 (Table 3). Thus, no case satisfied both the expected conditions.

**Table 3.** Statistical Analysis of the Lightness Distance (ΔL*) and Chromaticity Distance (ΔC*) Between Two Perceived Colours Among Groups 1, 2, and 3.

		Case 1	Case 2	Case 3	Case 4	Case 5
ΔL*	Group 1	27.8 ± 6.9	30.1 ± 7.2	14.6 ± 4.5	34 ± 5.8	28.1 ± 11.5
	Group 2	26.6 ± 10.8	23.6 ± 9.8	23.3 ± 8.9	33.6 ± 10.8	26 ± 8.6
	Group 3	20.3 ± 9.0	28.5 ± 7.3	15 ± 7	31.2 ± 5.9	14 ± 11.6
	*p* value	.1507	.033	<.001	.431	.001
	Post-hoc		1 > 2	1 < 2		1,2 > 3
ΔC*	Group 1	50.4 ± 13.7	35.4 ± 11.1	21.1 ± 8.5	28.2 ± 8.1	21.7 ± 7.3
	Group 2	51.8 ± 15.8	38.1 ± 12.5	29 ± 8	30.9 ± 8.1	26.9 ± 8.2
	Group 3	67.4 ± 9.7	49.3 ± 8.4	31.7 ± 4.1	44.4 ± 8.2	32.7 ± 8.9
	*p* value	.0165	.035	<.001	<.001	<.001
	Post-hoc	1,2 < 3	1,2 < 3	1 < 2,3	1,2 < 3	1 < 3

ΔL* in group 3 decreased in cases 1, 4, and 5, but Kruskal–Wallis analysis with Bonferroni post-hoc test shows that this was statistically significant only in Case 5. In all cases, ΔC* in group 3 increased, but this was statistically significant only in cases 1, 2, and 4.

## Discussion

In this study, we suggest that individual differences in colour perception are due to the characteristics of the two pixel colours in each object; that is, two colours are low saturated and are complementary to each other or are related in a complementary way. In addition, we propose that in these ambiguous photographs, there are differences in the estimation of the illuminant colour, and one of the two complementary colours must be estimated as the illuminant hue. Thus, people perceive the object's colours as being biased in complementarily different directions, and then perceive different pixel clusters as chromatic and achromatic, respectively. In addition, chromatic colours that are perceived differently among people tend to be complementary to each other.

Even if the illumination colours estimated by people are not different, the difference in their saturation and lightness may result in differences in colour perception. In Case 3, both pixel clusters in the image were shaded green, and the line connecting the two clusters passed through the center of the a* and b* planes. As represented in Figure 5, individual differences in colour perception were distinct along the a* axis, red–green distribution. If the colour that is perceived as achromatic is assumed to be an illuminant colour, the only possible illumination colour that could have been estimated was green. However, there was a different threshold for the degree in saturation and lightness to which the green signal was estimated as an illuminant in the object. In group 1, among the two low-saturated greens, green with relatively high saturation and high lightness was perceived as achromatic. In group 2, green with relatively low saturation and low lightness was perceived as achromatic. Thus, individual differences in estimating the contribution of illumination likely led to different perceptions of the two green pixel clusters in this case as well.

Indeed, the judgment of the illuminant colour between two complementary colours, where personal differences occurred among people, might be very complex and might depend on subjective assumptions regarding the brightness in these ambiguous photographs. There was a distinct difference in lightness perception of the subjects among respondents in our study, especially between groups 1 and 2. Moreover, there was a difference in colour perception between these groups. The important role of lightness in chromaticity perception of our cases is also in line with previous studies about “The Dress”. Considering the luminance–chromaticity distribution in natural daylight locus, most studies explained the reason why the achromatic settings depending on individual brightness sensitivity showed the opposite trend to the illumination matches in “The Dress” photograph (Aston & Hurlbert, 2017; Winkler et al., 2015; Witzel et al., 2017a). Generally, the colour of illumination is optimized to a dark and cold colour when there is an object in the shadow or indirect lighting. On the contrary, the colour of illumination is optimized to a bright and warm colour when the object is in direct light under the sun. Thus, the assumption of low brightness is linked to the estimation of darker and colder illumination in the scene, and in turn to a “bright and warm” match in colour perception. The higher brightness assumption is linked to brighter and warmer estimation of illuminant and in turn to a “dark and cold” match in colour perception (Aston & Hurlbert, 2017; Winkler et al., 2015; Witzel et al., 2017a). Thus, when people estimated the illuminant conditions considering the origin of the natural daylight, people should perceive the object's colours in additional ambiguous photographs within two colour categories, “dark achromatic and cold chromatic’ or “bright achromatic and warm chromatic.” Applying this idea to our cases, which caused individual differences in colour perception, cases 1, 2, 3, and 4 also showed consistent results with their proposal. In these cases, the object colours were matched to “bright and warm” colours in group 1 and to “dark and cold” colours in group 2.

However, in Case 5, the results differed from their proposal. Group 1 perceived the lightness of the object as high but the chromaticity as being biased toward the cold colour (blue–green) direction. In contrast, group 2 perceived the lightness of the jacket as low and the chromaticity as being biased toward the warm colour (yellow–red) direction. Thus, a new proposal is needed to explain the colour perception in these ambiguous photographs, including Case 5, where people might have estimated the atypical illumination condition in the colour perception process.

Interestingly, the different role of brightness balancing in estimating an illuminant and colour perception in these ambiguous photographs is reminiscent of the so-called Helson–Judd effect. It is known that the colour appearance modes are determined by the luminance relationship between an object and its surroundings (Kuriki, 2015; Pridmore, 2009). In addition, we propose that group 2, which perceived the object's lightness as low, might assess the object as being darker than the surroundings. They perceived the object's colours using the surface-colour mode based on estimating the darker pixel cluster as the illuminant colour. In contrast, group 1, which perceived the object’ lightness as high, might assess the object to be lighter than the surroundings, whereby it appeared to be self-luminous. Additionally, people perceived the object's colours based on estimating the brighter pixel cluster as the achromatic point in the scene. These assumptions are completely coherent with individual differences in colour perception of these ambiguous photographs and with the principles of colour constancy. However, the detailed characteristics of these underlying mechanisms remain unclear.

Hence, in group 3 of all cases, people perceived both pixel clusters as chromatic colours in these ambiguous photographs. This is not explained by illuminant estimation depending on brightness balance. Accordingly, it was expected that they might obtain colour constancy by relying on brightness-independent strategies, such as colour contrast. Thus, we expected that the chromaticity distance between two perceived colours would be prominent, and the lightness distance would be small in this group. However, the results were disappointing. We think that the inaccuracy of the test was due to an imbalance in the number of participants. If further experiments are conducted with more participants, we expect to know whether our hypothesis, that people in group 3 perceive colours in these ambiguous pictures by colour contrast and not by illuminant estimation based on brightness sensitivity, is correct.

## Supplementary Material

Supplementary material
